# Supplementary material for: Developing a Novel and Optimized Yeast Model for Human VDAC Research
Source: Int J Mol Sci. 2024 Dec 3;25(23):13010. doi: 10.3390/ijms252313010 (PMC11641349; doi:10.3390/ijms252313010)
Supplement: Supplementary file 1 [file ijms-25-13010-s001.zip › ijms-3313469-supplementary.pdf]

Supplementary File S1

Developing a novel and optimized yeast model for human VDAC research

Martyna Baranek-Grabińska, Wojciech Grabiński, Deborah Musso, Andonis Karachitos, Hanna Kmita

Sequences of repair DNA used in the CRISPR/Cas9 + homology-directed repair (HDR) method. Sequences from yeast genomic DNA upstream and downstream of the *POR1* open reading frame are shown in blue. Codon changes from cysteine to alanine are shown in green.

por1::hVDAC1

TTCCAACAAGTTTAATGGTCAGAATGGGCGCTTGTCGCGTGCCAGATCGGGGTTCAATTCCCCGTCGC
GGAGATTTTTTGCCGCACGTTTCCGCGTCAGACGAGCTTTTTTCACGGTTCCGGTGCTTTTTGCCGGC
TGCGGCCCAATCAAACACCGCCATTTCGGCCGTTCCTCAACAAAGAACCCCTTTTATAGCCAGCAGAG
CACGAGTTGATCTACTATATAACTACCCCCAACTCGTTCCACTACTCATTAGTGCTACGGATTCTCCC
AACACGAAACAGCCAAGCGTACCCAAAGCAAAAATCAAACCAACCTCTCAACAATGGCTGTTCCACCA
ACTTACGCTGACTTGGGTAAGTCTGCTAGAGACGTTTTCACTAAGGGTTACGGTTTCGGTTTGATCAA
GTTGGACTTGAAGACTAAGTCTGAAAACGGTTTGGAATTCACTTCTTCTGGTTCTGCTAACACTGAAA
CTACTAAGGTTACTGGTTCTTTGGAAACTAAGTACAGATGGACTGAATACGGTTTGACTTTCACTGAA
AAGTGGAACACTGACAACACTTTGGGTACTGAAATCACTGTTGAAGACCAATTGGCTAGAGGTTTGAA
GTTGACTTTCGACTCTTCTTTCTCTCCAAACACTGGTAAGAAGAACGCTAAGATCAAGACTGGTTACA
AGAGAGAACACATCAACTTGGGTTGTGACATGGACTTCGACATCGCTGGTCCATCTATCAGAGGTGCT
TTGGTTTTGGGTTACGAAGGTTGGTTGGCTGGTTACCAAATGAACTTCGAAACTGCTAAGTCTAGAGT
TACTCAATCTAACTTCGCTGTTGGTTACAAGACTGACGAATTCCAATTGCACACTAACGTTAACGACG
GTACTGAATTCGGTGGTTCTATCTACCAAAAGGTTAACAAGAAGTTGGAAACTGCTGTTAACTTGGCT
TGGACTGCTGGTAACTCTAACACTAGATTCGGTATCGCTGCTAAGTACCAAATCGACCCAGACGCTTG
TTTCTCTGCTAAGGTTAACAACTCTTCTTTGATCGGTTTGGGTTACACTCAAACTTTGAAGCCAGGTA
TCAAGTTGACTTTGTCTGCTTTGTTGGACGGTAAGAACGTTAACGCTGGTGGTCACAAGTTGGGTTTG
GGTTTGGAATTCCAAGCTTGAACGTATATATCTAATATATATATGTTCACTATATACCATATATGTGC
TCGTTCTTGTTTCCATTTTTCTACTTGATCTTTAGACTTGTTGTTTTATTTATACAAATATATTAATG
ACAGAAAATATTCTTTGTAACGGCTGGTTCTCATAATCATATTGGTTGCTTGGCAGTGAGAATAGACT
CACTTCCTGACTCATCATCACTTCCTTCCGCAGCCTCTAGCTCATTCAAATACAAGCAGTGTTTAGCA
AAGCCAGGGATAACGTTTCTCGGTATTTTCAGATTGGTTTCGAACATT

por1::hVDAC2

TTCCAACAAGTTTAATGGTCAGAATGGGCGCTTGTCGCGTGCCAGATCGGGGTTCAATTCCCCGTCGC
GGAGATTTTTTGCCGCACGTTTCCGCGTCAGACGAGCTTTTTTCACGGTTCCGGTGCTTTTTGCCGGC
TGCGGCCCAATCAAACACCGCCATTTCGGCCGTTCCTCAACAAAGAACCCCTTTTATAGCCAGCAGAG
CACGAGTTGATCTACTATATAACTACCCCCAACTCGTTCCACTACTCATTAGTGCTACGGATTCTCCC
AACACGAAACAGCCAAGCGTACCCAAAGCAAAAATCAAACCAACCTCTCAACAATGGCTACTCACGGT
CAAACTTGTGCTAGACCAATGTGTATCCCACCATCTTACGCTGACTTGGGTAAGGCTGCTAGAGACAT
CTTCAACAAGGGTTTCGGTTTCGGTTTGGTTAAGTTGGACGTTAAGACTAAGTCTTGTTCTGGTGTTG
AATTCTCTACTTCTGGTTCTTCTAACACTGACACTGGTAAGGTTACTGGTACTTTGGAAACTAAGTAC
AAGTGGTGTGAATACGGTTTGACTTTCACTGAAAAGTGGAACACTGACAACACTTTGGGTACTGAAAT
CGCTATCGAAGACCAAATCTGTCAAGGTTTGAAGTTGACTTTCGACACTACTTTCTCTCCAAACACTG
GTAAGAAGTCTGGTAAGATCAAGTCTTCTTACAAGAGAGAATGTATCAACTTGGGTTGTGACGTTGAC
TTCGACTTCGCTGGTCCAGCTATCCACGGTTCTGCTGTTTTCGGTTACGAAGGTTGGTTGGCTGGTTA
CCAAATGACTTTCGACTCTGCTAAGTCTAAGTTGACTAGAAACAACTTCGCTGTTGGTTACAGAACTG
GTGACTTCCAATTGCACACTAACGTTAACGACGGTACTGAATTCGGTGGTTCTATCTACCAAAAGGTT
TGTGAAGACTTGGACACTTCTGTTAACTTGGCTTGGACTTCTGGTACTAACTGTACTAGATTCGGTAT
CGCTGCTAAGTACCAATTGGACCCAACTGCTTCTATCTCTGCTAAGGTTAACAACTCTTCTTTGATCG
GTGTTGGTTACACTCAAACTTTGAGACCAGGTGTTAAGTTGACTTTGTCTGCTTTGGTTGACGGTAAG
TCTATCAACGCTGGTGGTCACAAGGTTGGTTTGGCTTTGGAATTGGAAGCTTGAACGTATATATCTAA
TATATATATGTTCACTATATACCATATATGTGCTCGTTCTTGTTTCCATTTTTCTACTTGATCTTTAG
ACTTGTTGTTTTATTTATACAAATATATTAATGACAGAAAATATTCTTTGTAACGGCTGGTTCTCATA
ATCATATTGGTTGCTTGGCAGTGAGAATAGACTCACTTCCTGACTCATCATCACTTCCTTCCGCAGCC
TCTAGCTCATTCAAATACAAGCAGTGTTTAGCAAAGCCAGGGATAACGTTTCTCGGTATTTTCAGATT
GGTTTCGAACATT

por1::hVDAC3

TTCCAACAAGTTTAATGGTCAGAATGGGCGCTTGTCGCGTGCCAGATCGGGGTTCAATTCCCCGTCGC
GGAGATTTTTTGCCGCACGTTTCCGCGTCAGACGAGCTTTTTTCACGGTTCCGGTGCTTTTTGCCGGC
TGCGGCCCAATCAAACACCGCCATTTCGGCCGTTCCTCAACAAAGAACCCCTTTTATAGCCAGCAGAG
CACGAGTTGATCTACTATATAACTACCCCCAACTCGTTCCACTACTCATTAGTGCTACGGATTCTCCC
AACACGAAACAGCCAAGCGTACCCAAAGCAAAAATCAAACCAACCTCTCAACAATGTGTAACACTCCA
ACATACTGCGACTTGGGCAAGGCTGCCAAGGATGTGTTCAACAAGGGTTACGGTTTTGGTATGGTTAA
GATAGACCTAAAGACCAAAAGTTGCTCCGGTGTCGAGTTTTCTACTTCTGGTCATGCTTACACCGATA
CCGGCAAGGCATCTGGAAACTTGGAAACCAAATACAAGGTGTGTAACTATGGTCTAACATTTACTCAA
AAGTGGAACACAGACAACACATTGGGCACCGAGATCAGCTGGGAAAACAAGTTGGCAGAGGGCTTGAA
GCTAACTCTGGACACGATTTTTGTTCCTAACACCGGCAAGAAGTCCGGTAAGTTGAAGGCTAGTTATA
AGCGAGATTGTTTCTCAGTTGGTTCTAACGTGGATATCGACTTCTCAGGTCCAACAATCTACGGTTGG
GCCGTCTTAGCCTTTGAGGGCTGGCTCGCCGGTTACCAAATGTCTTTCGACACCGCAAAGTCAAAACT
ATCCCAAAACAACTTCGCCCTGGGTTACAAGGCTGCCGATTTCCAATTGCATACGCATGTCAATGACG
GTACGGAGTTTGGTGGCTCCATTTATCAGAAGGTCAACGAAAAAATCGAAACTTCTATCAATTTGGCT
TGGACCGCCGGTTCCAATAACACTAGATTTGGTATTGCTGCCAAATATATGCTGGACTGTCGCACATC
CTTGTCCGCCAAAGTCAACAACGCCTCTTTGATCGGCTTGGGTTACACACAAACCTTAAGACCTGGCG
TCAAATTAACTTTGTCTGCTTTGATCGACGGTAAGAACTTTAGCGCCGGTGGCCACAAGGTCGGTCTA
GGTTTCGAATTGGAAGCATGAACGTATATATCTAATATATATATGTTCACTATATACCATATATGTGC
TCGTTCTTGTTTCCATTTTTCTACTTGATCTTTAGACTTGTTGTTTTATTTATACAAATATATTAATG
ACAGAAAATATTCTTTGTAACGGCTGGTTCTCATAATCATATTGGTTGCTTGGCAGTGAGAATAGACT
CACTTCCTGACTCATCATCACTTCCTTCCGCAGCCTCTAGCTCATTCAAATACAAGCAGTGTTTAGCA
AAGCCAGGGATAACGTTTCTCGGTATTTTCAGATTGGTTTCGAACATT

por1::hVDAC3ΔCys

TTCCAACAAGTTTAATGGTCAGAATGGGCGCTTGTCGCGTGCCAGATCGGGGTTCAATTCCCCGTCGC
GGAGATTTTTTGCCGCACGTTTCCGCGTCAGACGAGCTTTTTTCACGGTTCCGGTGCTTTTTGCCGGC
TGCGGCCCAATCAAACACCGCCATTTCGGCCGTTCCTCAACAAAGAACCCCTTTTATAGCCAGCAGAG
CACGAGTTGATCTACTATATAACTACCCCCAACTCGTTCCACTACTCATTAGTGCTACGGATTCTCCC
AACACGAAACAGCCAAGCGTACCCAAAGCAAAAATCAAACCAACCTCTCAACAATGGCGAACACTCCA
ACATACGCGGACTTGGGCAAGGCTGCCAAGGATGTGTTCAACAAGGGTTACGGTTTTGGTATGGTTAA
GATAGACCTAAAGACCAAAAGTGCGTCCGGTGTCGAGTTTTCTACTTCTGGTCATGCTTACACCGATA
CCGGCAAGGCATCTGGAAACTTGGAAACCAAATACAAGGTGGCGAACTATGGTCTAACATTTACTCAA
AAGTGGAACACAGACAACACATTGGGCACCGAGATCAGCTGGGAAAACAAGTTGGCAGAGGGCTTGAA
GCTAACTCTGGACACGATTTTTGTTCCTAACACCGGCAAGAAGTCCGGTAAGTTGAAGGCTAGTTATA
AGCGAGATGCGTTCTCAGTTGGTTCTAACGTGGATATCGACTTCTCAGGTCCAACAATCTACGGTTGG
GCCGTCTTAGCCTTTGAGGGCTGGCTCGCCGGTTACCAAATGTCTTTCGACACCGCAAAGTCAAAACT
ATCCCAAAACAACTTCGCCCTGGGTTACAAGGCTGCCGATTTCCAATTGCATACGCATGTCAATGACG
GTACGGAGTTTGGTGGCTCCATTTATCAGAAGGTCAACGAAAAAATCGAAACTTCTATCAATTTGGCT
TGGACCGCCGGTTCCAATAACACTAGATTTGGTATTGCTGCCAAATATATGCTGGACGCGCGCACATC
CTTGTCCGCCAAAGTCAACAACGCCTCTTTGATCGGCTTGGGTTACACACAAACCTTAAGACCTGGCG
TCAAATTAACTTTGTCTGCTTTGATCGACGGTAAGAACTTTAGCGCCGGTGGCCACAAGGTCGGTCTA
GGTTTCGAATTGGAAGCATGAACGTATATATCTAATATATATATGTTCACTATATACCATATATGTGC
TCGTTCTTGTTTCCATTTTTCTACTTGATCTTTAGACTTGTTGTTTTATTTATACAAATATATTAATG
ACAGAAAATATTCTTTGTAACGGCTGGTTCTCATAATCATATTGGTTGCTTGGCAGTGAGAATAGACT
CACTTCCTGACTCATCATCACTTCCTTCCGCAGCCTCTAGCTCATTCAAATACAAGCAGTGTTTAGCA
AAGCCAGGGATAACGTTTCTCGGTATTTTCAGATTGGTTTCGAACATT
